# Radiomics Analysis of Contrast-Enhanced CT Predicts Survival in Clear Cell Renal Cell Carcinoma

**DOI:** 10.3389/fonc.2021.671420

**Published:** 2021-06-25

**Authors:** Lei Yan, Guangjie Yang, Jingjing Cui, Wenjie Miao, Yangyang Wang, Yujun Zhao, Ning Wang, Aidi Gong, Na Guo, Pei Nie, Zhenguang Wang

**Affiliations:** ^1^ Department of Positron Emission Tomography-Computed Tomography (PET-CT) Center, The Affiliated Hospital of Qingdao University, Qingdao, China; ^2^ Scientific Research Department, Huiying Medical Technology Co., Ltd., Beijing, China; ^3^ Department of Radiology, Shandong Provincial Hospital, Jinan, China; ^4^ Department of Radiology, The Affiliated Hospital of Qingdao University, Qingdao, China

**Keywords:** radiomics, Rad-score, nomogram, clear cell renal cell carcinoma, overall survival

## Abstract

**Purpose:**

To develop and validate the radiomics nomogram that combines clinical factors and radiomics features to estimate overall survival (OS) in patients with clear cell renal cell carcinoma (ccRCC), and assess the incremental value of radiomics for OS estimation.

**Materials and Methods:**

One hundred ninety-four ccRCC cases were included in the training cohort and 188 ccRCC patients from another hospital as the test cohort. Three-dimensional region-of-interest segmentation was manually segmented on multiphasic contrast-enhanced abdominal CT images. Radiomics score (Rad-score) was calculated from a formula generated *via* least absolute shrinkage and selection operator (LASSO) Cox regression, after which the association between the Rad-score and OS was explored. The radiomics nomogram (clinical factors + Rad-score) was developed to demonstrate the incremental value of the Rad-score to the clinical nomogram for individualized OS estimation, which was then evaluated in relation to calibration and discrimination.

**Results:**

Rad-score, calculated using a linear combination of the 11 screened features multiplied by their respective LASSO Cox coefficients, was significantly associated with OS. Calibration curves showed good agreement between the OS predicted by the nomograms and observed outcomes. The radiomics nomogram presented higher discrimination capability compared to clinical nomogram in the training (C-index: 0.884; 95% CI: 0.808–0.940 *vs.* 0.803; 95% CI: 0.705–0.899, P < 0.05) and test cohorts (C-index: 0.859; 95% CI: 0.800–0.921 *vs.* 0.846; 95% CI: 0.777–0.915, P < 0.05).

**Conclusions:**

The radiomics nomogram may be used for predicting OS in patients with ccRCC, and radiomics is useful to assist quantitative and personalized treatment.

## Introduction

Renal cell carcinoma (RCC) is one of the most common urological malignancies that accounts for approximately 3.8% of all human cancers (1). Approximately 300,000 patients are diagnosed with RCC every year, resulting in 140,000 RCC-related deaths (2). The overall 5-year survival rate for the majority of patients (65%) diagnosed with localized RCC is 93%, whereas for those with lymph node metastasis or distant metastasis is 66 and 12%, respectively (3).

Clear cell renal cell carcinoma (ccRCC) constitutes the highest proportion (90%) of all diagnosed RCC and is the subtype with the poorest prognosis. In addition, ccRCC is associated with high metastatic potential (4). It is essential to find accurate predictive information for valid prognosis assessment, treatment planning, and implementation of surveillance strategies. Due to the lack of reliable biomarkers, prognostic prediction is primarily based on the combination of stage, grade, and histology (5). The conventional prognostic evaluation relies on the American Joint Committee on Cancers (AJCC) tumor-node-metastasis (TNM) staging system (6). However, the accuracy of the prognostic system for individualized prediction is limited (7). The prognostic models—Memorial Sloan Kettering Cancer Center [MSKCC]; Stage, Size, Grade, and Necrosis [SSIGN]; and the University of California at Los Angeles Integrated Staging System [UISS]—which include more clinical and pathological factors, have been proposed to guide the follow-up in RCC patients, including ccRCC (8–10). However, a prospective study that evaluated 1,647 patients with RCC reported that the above models have a slightly better prediction efficiency compared to the TNM staging system (5). Furthermore, the predictive ability of these models was instable after 2 years after diagnosis. Consequently, more accurate prediction models are needed to achieve precise and individualized prognostic evaluation.

Radiomics is a relatively new approach that extracts features from multimodality medical images using data-characterization algorithms (11). Over the last decade, radiomics features have been applied as imaging biomarkers for prognosis, staging, and prediction of cancer (12). The radiomics approach has been successfully applied to predict metastasis, recurrence, and other clinical outcomes of lung cancer, breast cancer, and colorectal cancer (13–15). To the best of our knowledge, the majority of previous radiomics studies related to RCC focused on the differentiation between malignant and benign renal lesions and the prediction of nuclear grading (16, 17), while only a few studies reported on radiomics-based research for prediction of overall survival (OS) in ccRCC. In this study, we investigated whether the radiomics features extracted from the enhanced CT images could be used to quantitatively assess the OS in patients with ccRCC.

## Materials and Methods

### Patients

**Figure 1:**
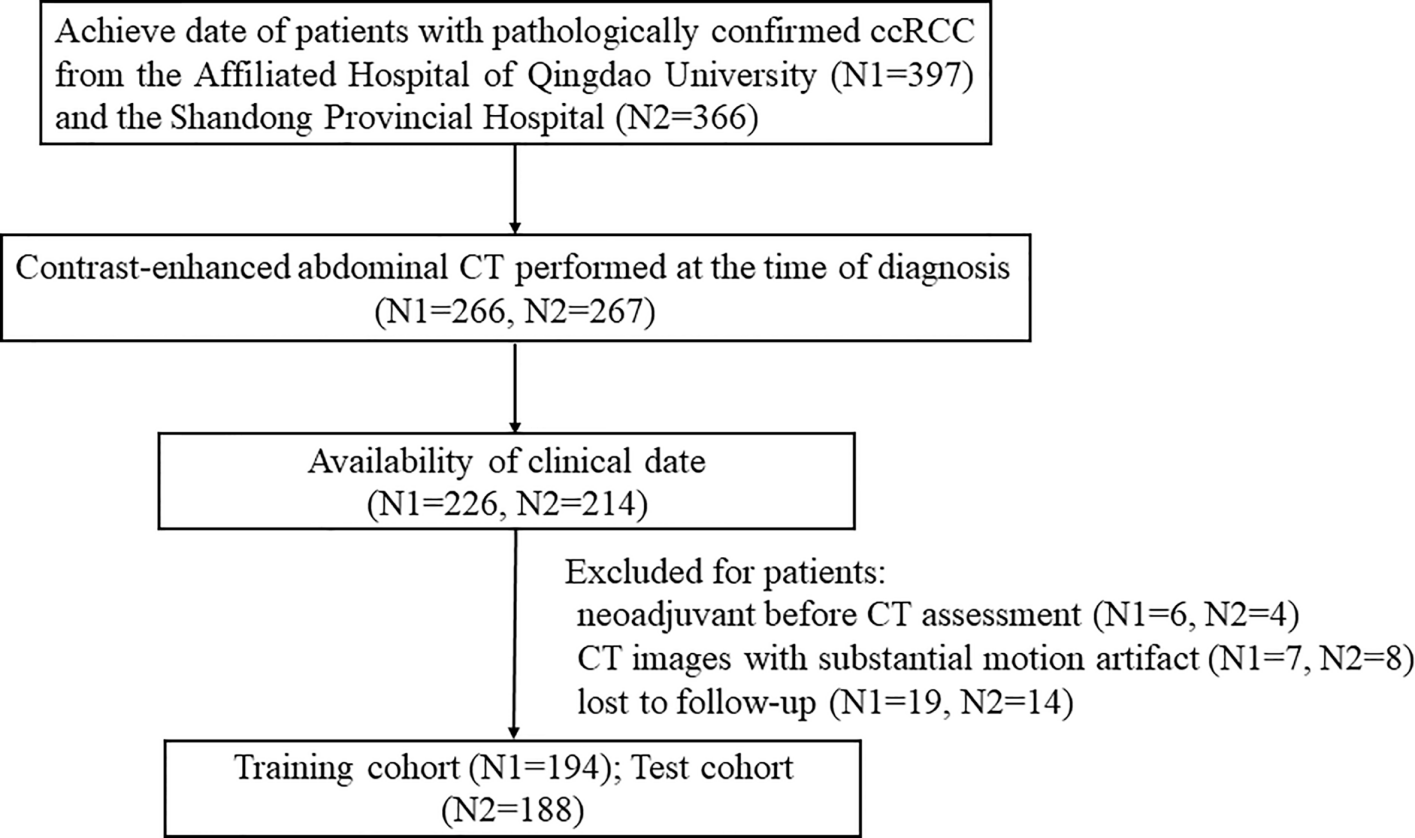
The patient recruitment pathway with inclusion and exclusion details.

Ethical approval for this retrospective study was obtained by the Ethics Committee of the Affiliated Hospital of Qingdao University. A total of 382 patients with ccRCC were enrolled in this two-center study. One hundred ninety-four patients from the Affiliated Hospital of Qingdao University (from May 2011 to December 2016) were collected as the training cohort, and 188 patients from Shandong Provincial Hospital (from June 2012 to May 2017) were collected as the independent test cohort. The detailed recruitment pathway for patients in this study is presented in **Figure 1**.

Three hundred fifty-one patients with TNM group I, II, and III were treated with radical or partial nephrectomy. Among 31 patients with TNM group IV, 29 patients underwent cytoreductive nephrectomy or tumors resection, and two patients with surgically unresectable tumors were treated with tissue sampling. After diagnosis, 20 out of the 31 patients received one or fewer systemic treatments (targeted therapy, immunotherapy, or chemotherapy).

The beginning of the follow-up was the date of diagnosis by pathological examination. Patients were reviewed every 6 to 12 months for the first 2 years, then every year after that. The review included clinical physical examination, chest X-ray, abdominal ultrasound, abdominal CT, or MRI. All data were collected in July 2019, and a follow-up phone call was conducted for patients who were not able to visit the hospital. The endpoint of the study was the time of death or the date of the last follow-up.

Baseline data consisted of age, sex, TNM group (I, II, III, or IV), Fuhrman grade, presence of histologic necrosis, hemoglobin (HB), neutrophil count (NE), lymphocyte count (LY), platelet count (PLT), blood urea nitrogen (BUN), creatinine (CREA), Eastern Cooperative Oncology Group Performance Status (ECOG-PS), and calculated neutrophil-lymphocyte ratio (NLR).

### CT Image Acquisition, Region-of-Interest Segmentation, and Radiomics Feature Extraction

CT scan protocols are explained in **Supplementary Methods**. The unenhanced abdominal scan was performed first. The enhanced scan was performed after injecting a 90–100 ml iodinated contrast agent (Ultravist 370, Bayer, Germany) into the antecubital vein at a flow rate of 2.5–3.0 ml/s. Images of corticomedullary phase (CMP), nephrographic phase (NP), and excretory phase (EP) were obtained at 30–35, 80–90, and 300–350 s after contrast injection, respectively.

Pre-treatment contrast-enhanced abdominal CT was exported in DICOM form from the picture archiving and communication system (PACS) workstation. Three-dimensional (3-D) ROI segmentation was manually performed using the ITK-SNAP software (Version 3.8.0, www.itksnap.org). Before feature extraction, image resampling and gray-level discretization were applied for the standardization of three-phase CT images. A total of 1,409 quantitative imaging features were extracted from each phase of CT images with the Radcloud platform (Huiying Medical Technology Co., Ltd). Radcloud platform was utilized to process the imaging and clinical data, as well as the spectra of radiomics analysis. The platform feature extraction is based on the “pyradiomics” package in Python (version 2.2.0, https://pyradiomics.readthedocs.io/). The features were grouped into three groups: (1) first-order statistics features describe the distribution of voxel intensities; (2) size- and shape-based features that reflect the size and shape of the region; (3) texture features that can quantify region heterogeneity differences. In addition, several filters were used to calculate the intensity and texture features on original images and derived images. The details of the radiomics features are shown in **Supplementary Methods**.

To obtain the inter-class correlation coefficient, ROIs of the 30 patients were segmented by two radiologists with 5 and 10 years of abdominal imaging experience, respectively. Then, the first radiologist completed the segmentation of the 30 ROIs after 2 weeks to obtain an intra-class correlation coefficient. To enhance the stability and reproducibility, radiomics features derived from the ROIs with both inter- and intra-class >0.75 were retained in the analysis and used in the following study. The first radiologist delineated the remaining ROIs.

### Feature Selection and Radiomics Score Calculation

The least absolute shrinkage and selection operator (LASSO) penalized Cox proportional hazards regression, which is appropriate for reducing high-dimensional data, was applied to select the optimal prognostic features in the training cohort. A formula was generated *via* a linear combination of the screened features multiplied by their respective LASSO Cox coefficients. Then, the formula was used to calculate the Rad-score of each individual. The median Rad-score was applied as a cutoff that stratified patients into the high-risk group with short survival time and the low-risk group with long survival time. The association of Rad-score with OS was estimated using the Kaplan-Meier survival analysis, and the difference in survival between the stratified subgroups was determined using the log-rank test.

### Rad-Score Assessment

Both 3-year and 5-year OS were described and analyzed in the training and test cohorts. The distribution of Rad-score in the 3-year survival group and the dead group was illustrated in a violin plot (box plot in the middle and a density plot on the side); Wilcoxon rank-sum test was used to analyze the significant difference. In addition, the prognostic accuracy of the Rad-score for the 3-year survival group and the dead group was assessed through the time-dependent receiver operating characteristic (ROC) analysis and the correlated area under the ROC curve (AUC). Rad-score for the 5-year survival group and the dead group was analyzed using the same processes.

### Development of the Clinical and Radiomics Nomograms

Clinical factors were assessed for their impact on OS by the univariable and multivariable Cox regression analysis in the training cohort. The clinical nomogram for probability prediction of 3- and 5-year OS was developed based on the multivariable Cox regression analysis. Independent prognostic clinical factors were selected through the last step, and Rad-score was used to develop the radiomics nomogram by the multivariable Cox regression analysis. The variables were no longer excluded in this section. The relative hazard ratio (HR) of each factor from the two nomograms was obtained simultaneously. The severity of multicollinearity among variables was detected using the Variance inflation factor (VIF) before the nomograms development regarding respective multivariable Cox regression. If VIF was <10, the multicollinearity was low.

### Performance of the Clinical and Radiomics Nomograms

To assess the performance of the clinical and radiomics nomograms, calibration and discrimination were performed in the training cohort and then validated in the test cohort. The calibration curve was used to indicate the agreement between the OS predicted by the nomograms and the observed outcomes after bias correction in 3- and 5-year OS. The Harrell concordance index (C-index) was measured to quantify the discrimination performance of the nomograms. To evaluate the goodness-of-fit of the nomograms, the Akaike information criterion (AIC) was generated. The discrimination capability of the two nomograms was compared to assess the incremental value of the Rad-score to the general clinical risk factors for an individualized assessment of OS in patients with ccRCC.

Additionally, the risk group, which was predicted by the nomogram with better discrimination capability, was used as a prediction factor to generate the Kaplan-Meier survival curves. If survival probability at 3 or 5 years predicted by the nomogram was <0.5, the patient was stratified into the nomogram-predicted (Nomo-predicted) high-risk group; if it was >0.5, the patient was stratified into the Nomo-predicted low-risk group. The difference between survival curves was assessed by using the log-rank test. The Kaplan-Meier survival curves were depicted by using actual survival status as prediction factor as well. The workflow of the study is shown in **Supplementary Figure 1**.

### Statistical Analysis

The statistical analysis was performed with SPSS version 24.0 and R software (version 3.3.3 https://www.r-project.org). The Student’s t-test, Chi-square test, or Non-parametric Mann-Whitney U test were applied to compare the differences in clinical factors between the training and test cohorts using SPSS software. Analysis and figure plots for the remaining data were performed using R software. A two-sided P < 0.05 was considered to be statistically significant.

## Results

### Clinical Factors and OS

By the time of the last follow-up, 60 patients (15.7%) died. The mean OS was 56 months, and the median OS was 59.0 months (interquartile range: 49.8–68.0 months) in the training cohort; the mean and median OS for the test cohort was 46 months and 43.5 months (interquartile range: 32.3–60.0 months), respectively. A significant difference in OS was observed between the two cohorts (P < 0.05), which was calculated based on the differences at follow-up time. No difference was found in sex, age, TNM group, presence of histologic necrosis, ECOG-PS, HB, LY, and BUN between the two cohorts (all P > 0.05), While differences in Fuhrman grade, NE, NLR, PLT, and CREA distribution were statistically significant (P < 0.05) **(** **Table 1** **)**.

**Table 1:** Baseline clinical date of the training and test cohorts.

Characteristic	Training cohort (n = 194)	Test cohort (n = 188)	P value
Sex			0.901^a^
Male	133	130	
Female	61	58	
Age (years)	55.94 ± 11.59	56.08 ± 10.96	0.906^b^
TNM group			0.597^a^
I	138	129	
II	29	29	
III	10	16	
IV	17	14	
Fuhrman grade			0.004^a^
I	11	16	
II	124	143	
III	50	27	
IV	9	2	
Necrosis			0.721^a^
Present	84	78	
Absent	110	110	
ECOG-PS			0.282^a^
0	105	112	
≥1	89	76	
Hemoglobin (g/L)	136.57 ± 19.797	138.70 ± 20.989	0.309^b^
Neutrophil count (10^9^/L)	3.746 ± 1.702	4.125 ± 1.751	0.032^b^
Lymphocyte count (10^9^/L)	1.972 ± 0.658	1.880 ± 0.660	0.171^b^
Neutrophil-lymphocyte ratio	2.200 ± 1.861	2.783 ± 3.183	0.029^b^
Platelet count (10^9^/L)	232.78 ± 65.225	248.09 ± 81.951	0.044^b^
Creatinine (umol/L)	88.626 ± 20.399	69.449 ± 18.189	<0.001^b^
Blood urea nitrogen (mmol/L)	6.268 ± 7.200	5.496 ± 1.829	0.154^b^
Overall Survival (month)			<0.001^c^
Median	59.0 (49.8–68.0)	43.5 (32.3–60.0)	
Mean	56.4 ± 17.3	46.0 ± 18.4	

ECOG-PS, Eastern Cooperative Oncology Group Performance Status.

a Chi-square test.

b Student’s t-test.

c Mann-Whitney U test.

### Feature Extraction, Selection, and Rad-score Calculation

**Figure 2:**
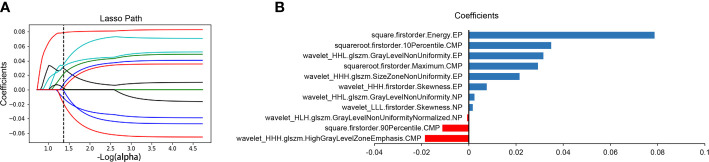
Radiomics feature selection and presentation. Radiomics feature selection using the LASSO Cox regression algorithm. LASSO regression coefficient profiles of survival-associated radiomics features **(A)**. A bar chart shows the contribution of selected features for radiomics signature construction **(B)**. The y-axis represents features that contribute to the signature. The x-axis represents corresponding coefficients in the LASSO Cox analysis. LASSO, least absolute shrinkage and selection operator; CMP, corticomedullary phase; NP, nephrographic phase; EP, excretory phase.

After excluding the subjective difference in ROI segmentation by observers, we only retained the repeatable and stable radiomics features with the inter- and intra-class correlation coefficients >0.75 and obtained 3,485 three-phase CT imaging features. After that, 11 optimal prognostic features were screened out through the LASSO Cox regression algorithm **(** **Figure 2A** **)**, and the radiomics signature was constructed. The signature calculation equation is depicted in **Supplementary Methods**. The bar chart below showed the contribution of selected features with their LASSO Cox regression coefficients for the signature construction **(** **Figure 2B** **)**.

**Figure 3:**
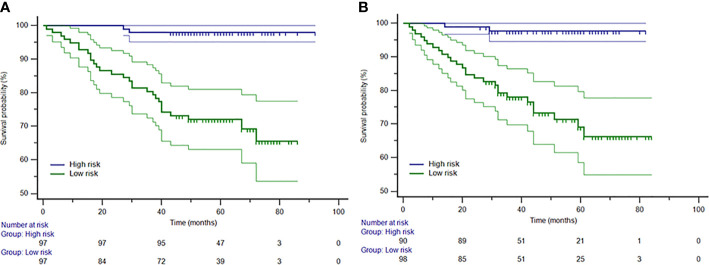
The Kaplan-Meier curves for patients in low-risk and high-risk groups in the training **(A)** and test **(B)** cohorts. The median Rad-score was −0.04481, which was applied as a cutoff that stratified patients into the high-risk group (Rad-score ≥ −0.04481) and the low-risk group (Rad-score < −0.04481).

The distribution of the Rad-score calculated by the equation for each patient is shown in **Supplementary Methods**. A distribution difference was observed in Rad-score between the training and test cohorts (P > 0.05). The cut-off value of the Rad-score was −0.04481. Consequently, patients were stratified into high-risk group (Rad-score ≥ −0.04481) and low-risk group (Rad-score < −0.04481). The Kaplan-Meier survival analysis showed a correlation between Rad-score and OS in the training cohort **(** **Figure 3A** **)**. The low-risk group has a longer OS compared to a high-risk group (P < 0.001, log-rank test). The same finding was demonstrated in the test cohort (**Figure 3B**; P < 0.001, log-rank test).

### Rad-Score Assessment

**Figure 4:**
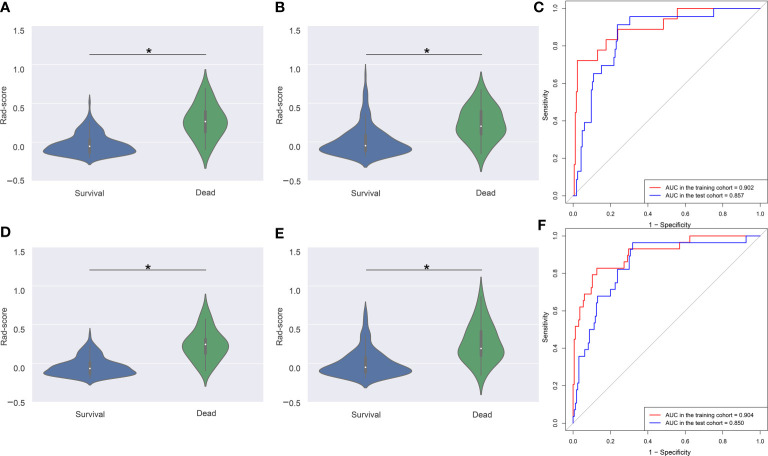
Violin plots and ROC curves of Rad-score in 3- and 5-year survival and dead groups. Violin plots show the distribution of Rad-score in 3-year survival and dead groups in the training **(A)** and test **(B)** cohorts. The median (central white dot), interquartile range (black box), and 95% confidence interval (vertical line) are shown in the middle. The color area represents a density plot on the side. ROC curves **(C)** for 3-year survival were determined to assess prognostic accuracy in the two cohorts. Violin plots **(D, E)** and ROC curves **(F)** in 5-year survival and dead groups. ROC, receiver operator characteristic. AUC, area under the curve. *P < 0.001.

Violin plots showed that the median Rad-score in 3- or 5-year dead groups were higher than that in 3- or 5-year survival groups in the training and test cohorts **(** **Figures 4A, B, D, E** **)**, and the distribution of Rad-score was significantly different (all P < 0.001). In addition, the AUC of the time-dependent ROC curves **(** **Figure 4C** **)** for 3-year OS in the training and test cohorts was 0.902 (95% CI: 0.851–0.940) and 0.857 (95% CI: 0.798–0.903), respectively. Satisfactory prognostic accuracy was achieved for the 5-year OS. AUC of the time-dependent ROC curves **(** **Figure 4F** **)** for 5-year OS in the training and test cohorts was 0.904 (95% CI: 0.854–0.942) and 0.850 (95% CI: 0.791–0.898), respectively. This data proved the discrimination accuracy of OS was reliable and robust when using Rad-score.

### Development of the Clinical and Radiomics Nomograms

**Figure 5:**
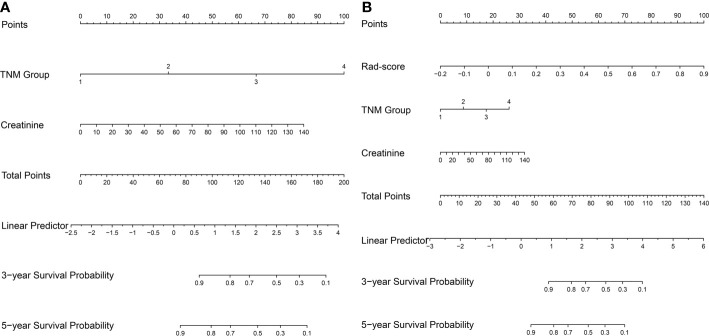
The nomogram for survival estimation. The clinical nomogram **(A)**, combing TNM group and creatinine. The radiomics nomogram **(B)**, combing Rad-score, TNM group, and creatinine.

Clinical factors were selected by the univariable and multivariable Cox regression analysis **(** **Table 2** **)**. The multivariable Cox regression showed that the TNM group and CREA were independent factors closely correlated with OS. So the clinical nomogram incorporating these two factors was developed **(** **Figure 5A** **)**. Both of these factors were negatively related to OS for patients (HR for TNM group: 2.912, 95% CI, 2.188–3.875; HR for CREA: 1.020, 95% CI, 1.004–1.036). The radiomics nomogram was developed with Rad-score, TNM group, and CREA by the multivariate Cox regression analysis **(** **Figure 5B** **)**. All three predictors were negatively correlated to OS (HR for Rad-score: 277.920, 95% CI, 26.039–2,966.297; HR for TNM group: 1.713, 95% CI, 1.149–2.552; HR for CREA: 1.014, 95% CI, 0.998–1.031).

**Table 2:** Uni- and multivariable COX regression analysis of predictors of OS.

Variable	Univariable Cox regression		Multivariable Cox regression	
	HR (95% CI)	P value	HR (95% CI)	P value
Sex	2.448 (0.940–6.377)	0.066	NA	
Age	1.044 (1.009–1.079)	0.012	1.026 (0.992–1.061)	0.141
TNM stage	2.908 (2.195–3.851)	<0.001	2.431 (1.709–3.459)	<0.001
Fuhrman grade	3.283 (2.037–5.291)	<0.001	1.188 (0.577–2.446)	0.640
Necrosis	2.513 (1.204–5.247)	0.014	1.595 (0.722–3.523)	0.248
ECOG-PS	2.371 (1.191–4.721)	0.014	1.235 (0.610–2.500)	0.557
Hemoglobin	0.964 (0.950–0.987)	<0.001	0.995 (0.974–1.016)	0.633
Neutrophil count	1.190 (1.040–1.361)	0.011	1.311 (0.84–2.044)	0.233
Lymphocyte count	0.358 (0.199–0.644)	0.001	0.395 (0.145–1.072)	0.068
Neutrophil-lymphocyte ratio	1.145 (1.055–1.243)	0.001	0.818 (0.55–1.217)	0.322
Platelet count	1.007 (1.002–1.012)	0.006	1.003 (0.998–1.007)	0.253
Creatinine	1.019 (1.006–1.033)	0.004	1.019 (1.003–1.036)	0.024
Blood urea nitrogen	1.001 (0.956–1.048)	0.954	NA	

NA, not available.

### Performance of the Clinical and Radiomics Nomograms

**Figure 6:**
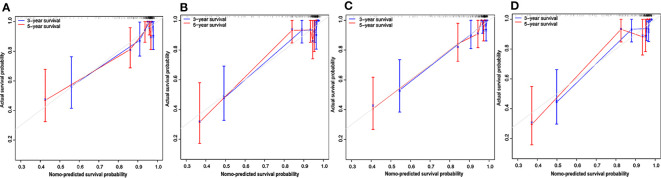
Calibration curves for the nomograms. Calibration curves for the clinical nomogram in the training **(A)** and test **(B)** cohorts. The y-axis indicates the actual probability of survival; the x-axis indicates the predicted probability of survival. The 45-degree gray line represents the ideal prediction; blue and red lines represent the performance of the clinical nomogram to predict 3- and 5-year survival, respectively. Calibration curves for the radiomics nomogram in the training **(C)** and test **(D)** cohorts. Nomo-predicted, nomogram-predicted.

The calibration curves depicted survival probability at 3 and 5 years after diagnosis, showing good agreement between survival probability predicted by the nomograms and observed outcomes in the training and test cohorts (**Figures 6A–D**). Integrating clinical factors and Rad-score, the radiomics nomogram obtained good discrimination performance with a C-index of 0.884 (95% CI: 0.808, 0.940), as well as higher discrimination capability compared with the clinical nomogram (P < 0.05) **(** **Supplementary Methods** **)**. The favorable result was confirmed in the test cohort, which implied the incremental value of the Rad-score for individual OS estimation. AIC and C-index estimates for the two nomograms are listed in **Table 3**.

**Table 3:** Performance of the nomograms.

	Training cohort		Test cohort	
	C-index (95% CI)	AIC	C-index (95% CI)	AIC
Clinical nomogram	0.803 (0.705–0.899)	263.26	0.846 (0.777–0.915)	233.65
Radiomics nomogram	0.884 (0.808–0.940)	243.35	0.859 (0.800–0.921)	234.25

**Figure 7:**
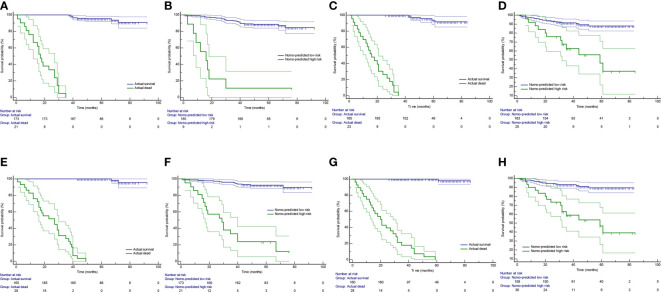
Kaplan-Meier survival curves in group stratification. Based on survival probability predicted by the radiomics nomogram, patients were stratified into the Nomo-predicted high-risk group (survival probability < 0.5) and the Nomo-predicted low-risk group (survival probability > 0.5). The Kaplan-Meier survival curves were depicted by using the actual survival status **(A)** and the Nomo-predicted risk group **(B)** at 3 years as prediction factor in the training cohort. **(C, D)** are corresponding figures in the test cohort. The Kaplan-Meier survival curves were depicted by using the actual survival status **(E)** and the Nomo-predicted risk group **(F)** at 5 years as prediction factor in the training cohort. **(G, H)** are corresponding figures in the test cohort. Areas between thin lines of the same color represent 95% confidence intervals.

The Kaplan-Meier survival curves were generated using a risk group based on survival probability at 3 or 5 years predicted by the radiomics nomogram as a prediction factor. A significant difference was confirmed between the stratified Nomo-predicted high-risk and low-risk groups in both the training and test cohorts (**Figures 7B, D, F, H**; P < 0.001, log-rank test). These results were consistent with the Kaplan-Meier survival analysis in the actual survival and dead groups (**Figures 7A, C, E, G**; P < 0.001, log-rank test).

## Discussion

ccRCC is the predominant pathological subtype of renal malignancy associated with aggressive behavior (high invasion and metastasis) and chemoresistance. Patients with ccRCC have the worse OS compared to those with other subtypes (18, 19). Hence it is essential to find an accurate prediction approach to improve prognosis and treatment in patient with ccRCC. In this study, we developed a radiomics nomogram by combining clinical factors and Rad-score for the prediction of OS in patients with ccRCC.

A number of previous studies have focused on identifying renal tumors and predicting nuclear grade by radiomics analysis, obtaining desirable results (20–22). Yet, so far, only a few studies have reported on prognostic prediction of RCC. Goh et al. assessed the texture parameters of 87 metastases in 39 RCC patients who received tyrosine kinase inhibitors (TKI) treatment and found that uniformity of texture was an independent predictive factor of time to progression (23). Another study showed that texture features consisting of the SD of pixel distribution histogram, entropy, and mean of positive pixels may be used to the prediction of OS for the patients with large RCCs (>7 cm; mean size, 9.9 cm) (24). These two studies indicated that texture analysis has the potential to predict the prognosis of RCC. More recently, Zeng et al. used integrative radiogenomics analysis (by analyzing contrast-enhanced CT images) for predicting molecular features and survival in ccRCC and found that these features could predict molecular subtypes, mutations, and prognosis of ccRCC patients (25). Moreover, Huang et al. suggested a radiomics model for predicting OS (5-year AUC = 0.775) in patients with the ccRCC model (26).

In this study, we discovered that the combination of radiomics and clinical data showed a higher predictive value than the clinical nomogram alone, thus suggesting it might be used to predict OS in patients with ccRCC. This study took valuable clinical factors into account. The TNM staging system is an internationally accepted system used to determine the disease stage, including RCC (27). Tumor grade is one of the strongest elements incorporated into prognostic models for patients with ccRCC (4). Histologic necrosis and ECOG-PS have shown to be independent risk factors for ccRCC patients in the SSIGN and UISS scoring systems, respectively (28). The value of laboratory examination and NLR was evaluated in the prognosis of malignant tumors such as kidney cancer (29, 30). In this study, TNM group and CREA were selected as the independent prognostic factors for survival. As for prognostic scoring systems, the MSKCC system was developed to define risk groups of patients by coalescing independent factors for survival prediction of metastatic RCC, while the UISS is an evidence-based system for predicting recurrence or metastases after surgical treatment in patients with localized or locally advanced RCC (31). The radiomics nomogram developed in this study was applied to both metastatic and locally ccRCC.

The evaluation of markers should depend on their ability to improve an already superior prediction model instead of on their P-value in multivariable analysis (32). Our results showed the radiomics nomogram performed better than the clinical nomogram, thus suggesting the incremental value of radiomics to OS prediction, and demonstrating that this new model is a useful method for outcome prognostication and treatment planning. In 2018, Meng et al. suggested that radiomics model combined with a clinicopathologic Cox model has a prognostic value for locally advanced rectal cancer (33). Another study extracted maximum and mean standard uptake values (SUVmax and SUVmean), total lesion glycolysis (TLG), metabolic tumoral volume (MTV), and texture features into Cox regression analysis in order to obtain prognostic model for identifying patients with more aggressive treatment (34). The importance of establishing comprehensive models was also reflected in the prognostic analysis of brain tumors, head-and-neck cancer, lung cancer, breast cancer, prostate cancer, liver cancer, and gastric cancer (12).

Ideally, an independent external validation dataset should be collected to test the results as with any biomarker analysis (35). Furthermore, developed models subsequently validated on an entirely new validation dataset from different centers can bolster its generalizability. Nonetheless, up to now, most of the external test dataset and the training dataset enrolled in studies were derived from the same center. Fortunately, an independent external test cohort from another hospital was assessed in this study to interpret the generalizability of the reported findings and correctly estimate the empirical error. As for the disparity of CT scanners arising from two institutes, image resampling, and gray-level discretization were implemented to standardize three-phase CT images, minimizing the impact of different scanning machines.

This study has a few limitations. First, this was a retrospective study, and the sample size was relatively small. Second, the clinical efficacy of our nomograms needs to be validated with the multicenter data. Last, patients with ccRCC were not classified into localized ccRCC, locally advanced ccRCC, and metastatic ccRCC groups according to the guidelines for stratified analysis. In this study, we did not compare the predictive ability between the radiomics nomogram and prognostic scoring system such as SSIGN, MSKCC. Thus, a large-scale prospective multicenter investigation is needed to further verify reported findings.

## Conclusions

In conclusion, we developed and validated a non-invasive predictive method for predicting the survival of ccRCC and identified radiomics as a useful biomarker for prognostic prediction. The radiomics analysis may facilitate quantitative and personalized treatment for ccRCC patients, although it still needs to be further validated before being widely applied in clinical practice.

## Data Availability Statement

The original contributions presented in the study are included in the article/**Supplementary Material**. Further inquiries can be directed to the corresponding authors.

## Author Contributions

Conception and design: LY and GY. Collection and assembly of data: LY, WM, YW, YZ, AG and NW. Development of methodology: LY, GY, JC, PN, and ZW. Data analysis and interpretation: GY, JC, WM, LY, and NG. Manuscript writing: LY, GY, and PN. All authors contributed to the article and approved the submitted version.

## Funding

This study was funded by the National Natural Science Foundation of China (81701688 and 81601527), the Natural Science Foundation of Shandong Province (ZR2017BH096 and ZR2017MH036), the Key Research and Development Project of Shandong Province (2018GSF118078), and the Postdoctoral Science Foundation of China (2018M642617).

## Conflict of Interest

Authors JC and NG were employed by company Huiying Medical Technology Co., Ltd.

The remaining authors declare that the research was conducted in the absence of any commercial or financial relationships that could be construed as a potential conflict of interest.

The reviewer QQ declared a shared affiliation with one of the authors, NW, to the handling editor at time of review.
