# Supplementary figures and images for: Radiomics Analysis of Contrast-Enhanced CT Predicts Survival in Clear Cell Renal Cell Carcinoma
Source: Front Oncol. 2021 Jun 25;11:671420. doi: 10.3389/fonc.2021.671420 (PMC8268016; doi:10.3389/fonc.2021.671420)

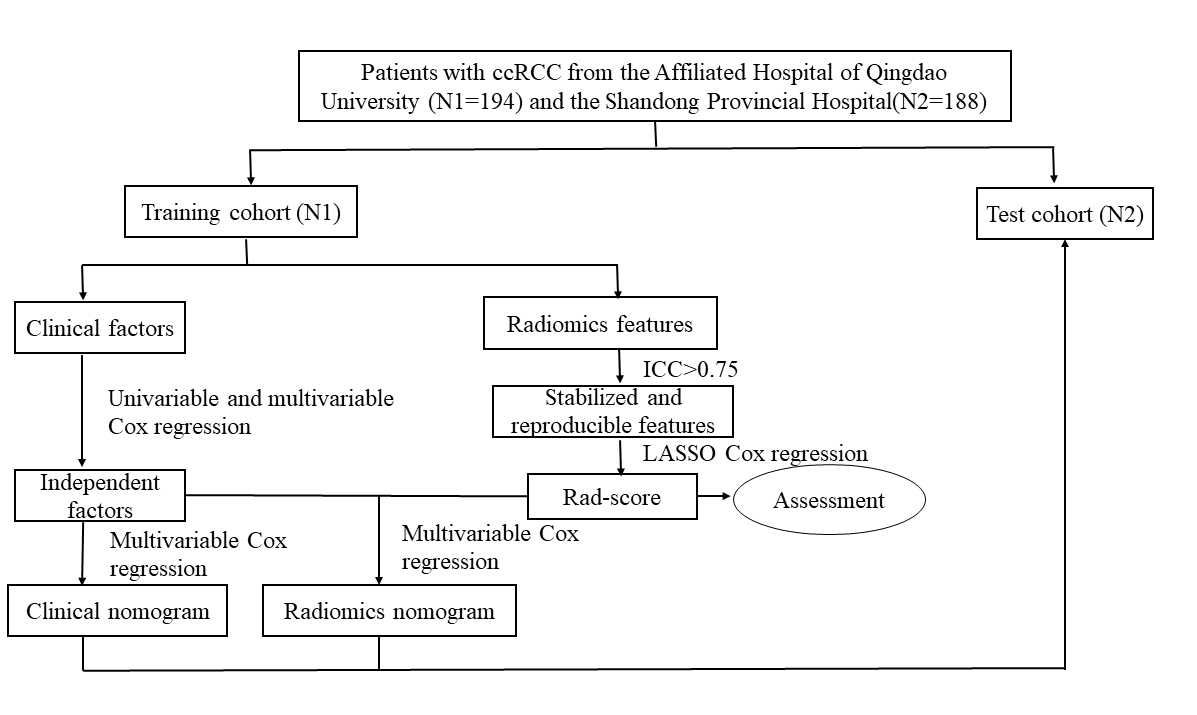

Supplement: Supplementary file 1 [file Image_1.tif]
