# Supplementary material for: Radiomics Analysis of Contrast-Enhanced CT Predicts Survival in Clear Cell Renal Cell Carcinoma
Source: Front Oncol. 2021 Jun 25;11:671420. doi: 10.3389/fonc.2021.671420 (PMC8268016; doi:10.3389/fonc.2021.671420)
Supplement: Supplementary file 2 [file Table_1.docx]

**Supplementary Table 1**

**CT scan protocols**

| **CT scanner** | **CT 64** | | | **CT 64** |
| --- | --- | --- | --- | --- |
| Scanner model | | Sensation 64 | Discovery 750 | |
| Manufacturer | | Siemens | General electric | |
| Gantry rotation time | | 0.5 | 0.5  120  250–400 mA (using automatic tube current modulation) | |
| Tube voltage (kV) | | 120 |  |  |
| Tube current | | 200 mAs |  |  |
| Detector collimation (mm) | | 64 × 0.6 | 64 × 0.625 | |
| Matrix | | 512 × 512 | 512 × 512 | |
| Pitch | | 1.0 | 1.375 | |
| Slice thickness (mm) | | 5 | 5 | |

s, second; kV, kilovolt; mA, milliampere; mm, millimeter

**The details of the radiomics features:**

A total of 1409 quantitative imaging features were extracted from CT images with Radcloud platform, these features can be grouped into three groups. (1) First-order statistics (n = 18), described the intensity information in the CT image region of interest, such as mean, standard deviation, variance, maximum, median, range, etc. (2) Size- and shape-based features (n = 14), which reflected the size and shape of the region, such as volume, surface area, compactness, aximum diameter, etc. (3) Texture features, which could quantify regional heterogeneity differences, such as gray-level co-occurrence matrix (GLCM, n = 24), gray-level size zone matrix (GLSZM, n = 16), gray-level dependence matrix (GLDM, n = 14), neighborhood gray-level dependence matrix (NGLDM, n=5), and gray-level run-length matrix (GLRLM, n = 16). Another 1302 features, including the first-order statistics and texture features derived from filter of the original images: logarithm, exponential, gradient, square, square root, local binary patterns (LBP), the wavelet transform decomposes the tumor area image into low-frequency components (L) or high-frequency components (H) in the three directions of the x, y, and z axes. Eight types of wavelet features were obtained and labeled as LLL, LLH, LHL, LHH, HLL, HLH, HHL, HHH according to their different decomposition orders.

**The signature calculation equation**

Rad_score = -0.0186* wavelet_HHH.glszm.HighGrayLevelZoneEmphasis.CMP-

0.0112* square.firstorder.90Percentile.CMP-

0.0007* wavelet_HLH.glszm.GrayLevelNonUniformityNormalized.NP+

0.0017* wavelet_LLL.firstorder.Skewness.NP+

0.0024* wavelet_HHL.glszm.GrayLevelNonUniformity.NP+

0.0076* wavelet_HHH.firstorder.Skewness.EP+

0.0215* wavelet_HHH.glszm.SizeZoneNonUniformity.EP+

0.0293* squareroot.firstorder.Maximum.CMP+

0.0316* wavelet_HHL.glszm.GrayLevelNonUniformity.EP+

0.0349* squareroot.firstorder.10Percentile.CMP+

0.0787* square.firstorder.Energy.EP

**Supplementary Table 2**

**The distribution of Rad-score calculated by the equation for each patient**

| **Training cohort** | | |  | **Test cohort** | | |
| --- | --- | --- | --- | --- | --- | --- |
| Patient ID | Radiomics score | Overall survival  (month) |  | Patient ID | Radiomics score | Overall survival (month) |
| 2428622 | -0.162510192 | 62 |  | 787458 | -0.156717147 | 26 |
| 2474571 | -0.171194183 | 50 |  | 774139 | 0.409555208 | 28 |
| 2459164 | 0.100852017 | 61 |  | 769984 | -0.098654714 | 28 |
| 2076266 | 0.142499255 | 80 |  | 765715 | -0.149853917 | 28 |
| 2041404 | -0.141528122 | 82 |  | 759833 | -0.085574018 | 29 |
| 2279982 | -0.08524062 | 69 |  | 759053 | -0.150435492 | 28 |
| 2419376 | -0.171912174 | 63 |  | 758907 | -0.155895103 | 29 |
| 2153456 | -0.082090796 | 76 |  | 755173 | 0.163811761 | 29 |
| 2219002 | 0.03187222 | 72 |  | 754866 | -0.13565261 | 29 |
| 2854181 | 0.031112527 | 47 |  | 739482 | -0.031665203 | 30 |
| 2631583 | 0.15550204 | 55 |  | 738530 | 0.00296458 | 30 |
| 2752956 | -0.140825706 | 51 |  | 737663 | 0.085198821 | 30 |
| 2405561 | -0.02592993 | 63 |  | 736756 | 0.195648397 | 31 |
| 2468069 | 0.230605517 | 61 |  | 735470 | -0.062467556 | 30 |
| 1188255 | -0.10099316 | 63 |  | 730675 | -0.139750791 | 31 |
| 2072719 | -0.02492226 | 57 |  | 725515 | -0.155314985 | 32 |
| 2342285 | -0.051014878 | 56 |  | 724058 | 0.473858425 | 8 |
| 2480915 | 0.020735904 | 61 |  | 723889 | -0.14411915 | 32 |
| 2520164 | -0.130267603 | 59 |  | 723782 | 0.142745422 | 32 |
| 1979488 | 0.11150086 | 46 |  | 722389 | 0.039148617 | 32 |
| 2393707 | -0.045594294 | 64 |  | 719558 | 0.348019493 | 32 |
| 2177893 | -0.149821246 | 74 |  | 711812 | -0.006316481 | 31 |
| 2125573 | -0.163783004 | 78 |  | 709555 | 0.471062922 | 6 |
| 1922786 | -0.157188358 | 86 |  | 706985 | 0.012708588 | 33 |
| 2729127 | -0.121776101 | 51 |  | 706411 | -0.124831716 | 31 |
| 2761346 | -0.146838395 | 49 |  | 706179 | 0.327650105 | 33 |
| 2712346 | -0.024220609 | 52 |  | 705507 | -0.174052571 | 29 |
| 2639400 | -0.014966263 | 55 |  | 696201 | 0.354069775 | 20 |
| 2539682 | -0.138184492 | 59 |  | 694369 | 0.122123386 | 21 |
| 2631361 | -0.167641776 | 55 |  | 693102 | 0.371376353 | 13 |
| 2222182 | 0.025451347 | 72 |  | 686399 | 0.030696055 | 34 |
| 2504792 | -0.143718841 | 59 |  | 684619 | 0.651345219 | 35 |
| 2378844 | -0.117045821 | 64 |  | 681156 | 0.090900156 | 35 |
| 2543536 | 0.019703303 | 59 |  | 679242 | -0.082190607 | 35 |
| 2291857 | -0.056351107 | 68 |  | 677247 | -0.155249597 | 35 |
| 2305808 | -0.134582743 | 67 |  | 671502 | -0.143803078 | 36 |
| 2211839 | 0.165185904 | 72 |  | 670666 | -0.050252874 | 36 |
| 2843375 | -0.187551722 | 47 |  | 666438 | 0.250704719 | 32 |
| 2130120 | -0.119778162 | 77 |  | 666407 | -0.035505933 | 36 |
| 2516453 | -0.125809136 | 60 |  | 663485 | 0.110733695 | 36 |
| 2763828 | 0.129853316 | 50 |  | 663460 | -0.097364977 | 36 |
| 2449494 | -0.095632394 | 62 |  | 662521 | -0.113183988 | 36 |
| 2566139 | -0.045243529 | 58 |  | 662514 | -0.169390879 | 36 |
| 2314590 | -0.148563912 | 67 |  | 662098 | 0.316151064 | 36 |
| 2535183 | -0.178194342 | 49 |  | 659414 | -0.088405383 | 37 |
| 2525053 | -0.006583821 | 59 |  | 653947 | 0.01433115 | 37 |
| 2261853 | -0.066963088 | 70 |  | 653143 | -0.122585795 | 37 |
| 2712432 | -0.178599266 | 52 |  | 650083 | 0.665462856 | 37 |
| 2738908 | -0.133512434 | 51 |  | 647546 | -0.047773413 | 38 |
| 2116757 | -0.188574508 | 55 |  | 646750 | 0.095624735 | 38 |
| 2130705 | 0.091682825 | 77 |  | 645431 | 0.113902779 | 38 |
| 2609866 | 0.063056228 | 56 |  | 638969 | -0.158361443 | 38 |
| 2664193 | -0.149591142 | 51 |  | 638920 | -0.101193521 | 38 |
| 2517197 | 0.161302743 | 60 |  | 637291 | 0.407805804 | 38 |
| 2793551 | -0.107883351 | 49 |  | 637052 | -0.099842916 | 38 |
| 2401436 | 0.158198069 | 63 |  | 632585 | -0.041419436 | 39 |
| 2825824 | -0.11178662 | 48 |  | 631994 | -0.102467317 | 14 |
| 2907411 | 0.002063825 | 45 |  | 630460 | -0.120436596 | 39 |
| 2984434 | -0.050242273 | 43 |  | 625471 | -0.063828981 | 39 |
| 2661426 | -0.044870034 | 54 |  | 624378 | -0.114777312 | 39 |
| 2138906 | -0.148576133 | 76 |  | 624246 | -0.134581156 | 39 |
| 1988147 | 0.236896305 | 86 |  | 622344 | -0.177510204 | 32 |
| 2649773 | -0.1571137 | 55 |  | 621845 | -0.047263364 | 39 |
| 2075371 | -0.188563083 | 80 |  | 621297 | -0.134313922 | 40 |
| 2306606 | -0.064242036 | 67 |  | 618207 | -0.1750744 | 40 |
| 2769790 | -0.106974329 | 50 |  | 617025 | -0.015613572 | 40 |
| 2393584 | 0.158375644 | 64 |  | 616159 | -0.143390207 | 40 |
| 2119767 | -0.08501295 | 78 |  | 614959 | -0.105807449 | 40 |
| 2770069 | -0.137197054 | 49 |  | 608138 | 0.058120762 | 16 |
| 2238231 | 0.102469282 | 71 |  | 598208 | 0.058747896 | 42 |
| 2277324 | -0.166414981 | 68 |  | 597597 | -0.074239237 | 42 |
| 2244738 | -0.071983119 | 68 |  | 592480 | -0.109343076 | 42 |
| 2316243 | -0.126546342 | 67 |  | 592167 | -0.134156823 | 42 |
| 2501892 | -0.144590488 | 60 |  | 591554 | -0.025055427 | 42 |
| 2912127 | -0.138279825 | 45 |  | 591338 | -0.152748717 | 29 |
| 2145969 | -0.128449569 | 76 |  | 591307 | -0.169090787 | 42 |
| 2745702 | 0.075249084 | 51 |  | 578882 | 0.04163474 | 43 |
| 2915676 | -0.080706493 | 45 |  | 572227 | 0.673820821 | 32 |
| 2133674 | -0.025878942 | 77 |  | 571360 | -0.117111396 | 44 |
| 2732486 | -0.128614889 | 51 |  | 567648 | 0.157620698 | 44 |
| 2874765 | -0.132890055 | 47 |  | 567047 | 0.251940239 | 32 |
| 2745572 | 0.081163894 | 51 |  | 566064 | -0.152045615 | 44 |
| 2065220 | -0.001543507 | 76 |  | 553145 | 0.116699266 | 42 |
| 2154068 | 0.030533746 | 76 |  | 552899 | 0.424828782 | 15 |
| 2238911 | -0.039902612 | 71 |  | 547969 | 0.060635125 | 44 |
| 2088019 | -0.013735584 | 73 |  | 546662 | 0.209455129 | 46 |
| 2566767 | -0.125552895 | 58 |  | 545968 | 0.052753217 | 10 |
| 2431702 | 0.137568859 | 62 |  | 535182 | -0.08531919 | 47 |
| 2158291 | -0.053140516 | 75 |  | 534543 | -0.187865502 | 47 |
| 2408648 | 0.011596146 | 63 |  | 534421 | 0.010379183 | 47 |
| 2543796 | -0.084165751 | 59 |  | 533101 | -0.094866356 | 47 |
| 2121759 | 0.051903646 | 78 |  | 526833 | -0.046456763 | 48 |
| 2653147 | -0.128101481 | 54 |  | 523728 | 0.251131565 | 48 |
| 2348481 | -0.136616059 | 66 |  | 518312 | -0.082405002 | 49 |
| 2897240 | -0.168707009 | 46 |  | 516180 | -0.164359453 | 49 |
| 2402666 | -0.153778578 | 63 |  | 515748 | 0.524872639 | 49 |
| 2494349 | -0.149036017 | 60 |  | 513910 | 0.023116875 | 49 |
| 2482326 | 0.104671141 | 54 |  | 513613 | 0.117795658 | 49 |
| 2512347 | -0.011638495 | 60 |  | 513564 | -0.05374333 | 49 |
| 2625569 | -0.002652464 | 55 |  | 506105 | -0.048401849 | 50 |
| 2166984 | -0.034895301 | 75 |  | 503504 | 0.583322337 | 35 |
| 2711794 | -0.131011935 | 52 |  | 497845 | -0.048468773 | 50 |
| 2933117 | -0.147675436 | 44 |  | 496399 | 0.140274511 | 27 |
| 2337401 | -0.147485027 | 66 |  | 484424 | -0.029029431 | 52 |
| 2591360 | 0.247295285 | 57 |  | 480661 | -0.110084892 | 52 |
| 2627308 | -0.04082153 | 55 |  | 480659 | -0.02777589 | 12 |
| 2303759 | -0.122102752 | 68 |  | 475747 | -0.05122242 | 52 |
| 2173154 | -0.112631931 | 74 |  | 464307 | -0.173662287 | 54 |
| 2018721 | -0.023783719 | 84 |  | 458876 | 0.651073753 | 44 |
| 2104418 | 0.218302987 | 79 |  | 458330 | -0.185938894 | 54 |
| 2109523 | -0.144813238 | 77 |  | 457995 | 0.099322251 | 54 |
| 2050251 | 0.368064826 | 76 |  | 456967 | -0.147397437 | 55 |
| 2166901 | -0.076029923 | 75 |  | 444820 | -0.102826329 | 55 |
| 2180899 | 0.01840498 | 74 |  | 440731 | 0.160141754 | 7 |
| 2189670 | -0.156377444 | 74 |  | 439973 | 0.039431032 | 55 |
| 2201142 | -0.0215027 | 73 |  | 437517 | -0.122082509 | 56 |
| 1643287 | -0.170546996 | 71 |  | 429817 | 0.408670986 | 21 |
| 2232132 | -0.168718674 | 71 |  | 429435 | -0.056914812 | 57 |
| 2265808 | 0.072509292 | 70 |  | 426958 | 0.140201284 | 57 |
| 2232735 | -0.044749952 | 69 |  | 425156 | -0.17820818 | 57 |
| 2143540 | -0.110555555 | 69 |  | 421636 | 0.077230118 | 2 |
| 2248388 | -0.086316646 | 68 |  | 420738 | 0.14083849 | 58 |
| 2305168 | -0.015403631 | 67 |  | 420542 | -0.148319089 | 58 |
| 2314449 | -0.116019702 | 67 |  | 415996 | 0.074080912 | 58 |
| 2335448 | -0.053813114 | 66 |  | 413472 | 0.181621217 | 3 |
| 2405861 | -0.181453175 | 64 |  | 411180 | 0.385959218 | 31 |
| 2405787 | -0.144331133 | 63 |  | 409850 | 0.109708826 | 51 |
| 2415149 | 0.004163434 | 63 |  | 409805 | -0.010676371 | 59 |
| 2422398 | 0.097545515 | 63 |  | 408374 | -0.048227611 | 59 |
| 2376426 | -0.056534758 | 63 |  | 406925 | 0.363605136 | 59 |
| 2407274 | -0.042681407 | 63 |  | 406448 | 0.149652252 | 59 |
| 2404627 | 0.226270926 | 63 |  | 406049 | 0.225926037 | 60 |
| 2442134 | 0.196779688 | 62 |  | 401169 | -0.057111506 | 60 |
| 2401575 | -0.066273344 | 62 |  | 400551 | -0.089477525 | 60 |
| 2462992 | -0.142775111 | 61 |  | 399471 | 0.071936252 | 60 |
| 2501657 | -0.070437397 | 60 |  | 393644 | 0.861730587 | 59 |
| 2437377 | 0.018474741 | 61 |  | 385555 | -0.150219605 | 61 |
| 2476884 | -0.073089283 | 61 |  | 372995 | -0.113650987 | 63 |
| 2535463 | 0.175027154 | 59 |  | 372904 | -0.054454469 | 37 |
| 2558139 | 0.164285527 | 58 |  | 368916 | -0.112381389 | 63 |
| 2561829 | 0.286012911 | 58 |  | 367221 | -0.118788853 | 63 |
| 2092263 | -0.034382829 | 57 |  | 361732 | 0.387282118 | 61 |
| 2640239 | -0.077754043 | 55 |  | 360078 | 0.133889674 | 64 |
| 2627411 | -0.110369539 | 55 |  | 358364 | 0.202628852 | 4 |
| 2640029 | -0.039765657 | 55 |  | 357625 | -0.102757701 | 64 |
| 2187623 | -0.005006414 | 55 |  | 354384 | -0.154844303 | 45 |
| 2643981 | -0.195973527 | 55 |  | 352972 | -0.151853354 | 65 |
| 2681754 | -0.15158265 | 54 |  | 345385 | -0.091109108 | 66 |
| 2663562 | -0.154073754 | 54 |  | 342861 | -0.170092013 | 67 |
| 2655678 | -0.13260112 | 53 |  | 337641 | 0.349056706 | 67 |
| 2696100 | -0.179802437 | 53 |  | 336140 | -0.070756947 | 67 |
| 2705102 | -0.072126846 | 52 |  | 334160 | 0.134935829 | 18 |
| 2592022 | -0.087195956 | 52 |  | 333667 | -0.102870846 | 68 |
| 2742886 | 0.093859222 | 51 |  | 332603 | -0.077425933 | 68 |
| 2747273 | -0.014899911 | 51 |  | 330559 | 0.07772109 | 65 |
| 2787508 | -0.039064176 | 49 |  | 322565 | 0.123735381 | 69 |
| 2789951 | 0.070045339 | 49 |  | 320279 | 0.110490216 | 69 |
| 2838841 | -0.052894771 | 48 |  | 320092 | -0.111173079 | 69 |
| 2858084 | -0.0483135 | 47 |  | 318060 | 0.049306323 | 70 |
| 2507488 | -0.011086758 | 60 |  | 313843 | -0.001888907 | 66 |
| 2520746 | -0.021341264 | 59 |  | 308471 | 0.042050078 | 71 |
| 1693083 | -0.075796468 | 92 |  | 306157 | 0.14809043 | 71 |
| 2023893 | -0.01144 | 83 |  | 304562 | -0.005955113 | 71 |
| 2038532 | 0.136844443 | 16 |  | 301782 | -0.128616481 | 71 |
| 2629327 | 0.367369582 | 15 |  | 297520 | -0.132619672 | 72 |
| 2220262 | 0.516101408 | 30 |  | 297171 | -0.037636714 | 72 |
| 2574813 | 0.440255568 | 3 |  | 296964 | -0.075397683 | 72 |
| 1929471 | 0.180876932 | 67 |  | 294685 | -0.06166388 | 72 |
| 2508248 | 0.312019797 | 17 |  | 291312 | 0.075325204 | 73 |
| 2245271 | 0.127758602 | 23 |  | 288493 | -0.113131373 | 73 |
| 2686945 | 0.198048646 | 40 |  | 287901 | -0.033500968 | 73 |
| 2204858 | -0.095324508 | 27 |  | 284994 | -0.107715326 | 73 |
| 2144930 | 0.300634938 | 35 |  | 284211 | 0.563879893 | 73 |
| 2433214 | 0.185101767 | 27 |  | 283901 | 0.082644891 | 74 |
| 2478545 | 0.181850938 | 30 |  | 278318 | 0.251100918 | 24 |
| 2945737 | -0.073219072 | 29 |  | 273222 | -0.046195675 | 72 |
| 2157926 | 0.258092271 | 19 |  | 273102 | -0.109583448 | 44 |
| 2337292 | -0.012278857 | 40 |  | 269891 | 0.157931719 | 75 |
| 2395024 | 0.244778153 | 6 |  | 260948 | 0.004152994 | 76 |
| 2558141 | 0.405860609 | 9 |  | 258717 | 0.052822472 | 77 |
| 3167186 | 0.693470251 | 12 |  | 241618 | 0.062546493 | 79 |
| 2162041 | 0.112891237 | 12 |  | 236014 | -0.151414798 | 80 |
| 2839442 | 0.293865036 | 40 |  | 221267 | -0.097127587 | 82 |
| 3221778 | 0.572391758 | 16 |  | 218777 | 0.232703542 | 82 |
| 3251407 | 0.296105402 | 18 |  | 214392 | 0.012579595 | 83 |
| 2677059 | 0.569519875 | 7 |  | 209767 | 0.399909463 | 84 |
| 2166961 | 0.035046265 | 43 |  | 201996 | 0.52578146 | 61 |
| 2062145 | 0.120409942 | 72 |  | 126661 | 0.01532194 | 67 |
| 1084354 | 0.169029937 | 39 |  |  |  |  |
| 2644673 | 0.128839551 | 37 |  |  |  |  |
| 2719896 | 0.272023396 | 29 |  |  |  |  |
| 2798570 | -0.009464145 | 38 |  |  |  |  |
| 1961879 | 0.046916402 | 1 |  |  |  |  |
| 2213518 | 0.263966199 | 49 |  |  |  |  |

**Supplementary Table 3**

Comparison of the performance of nomograms

|  | **Training cohort** | | |  |  | **Test cohort** | | | |
| --- | --- | --- | --- | --- | --- | --- | --- | --- | --- |
|  | **C-index (95%CI)** | **P value** | |  |  | **C-index (95%CI)** | | **P value** | |
| Clinical nomogram  VS | 0.803(0.705-0.899) |  | 0.035^a^ | | | | 0.846 (0.777-0.915)  VS | | 0.005^a^ |
| Radiomics nomogram | 0.884(0.808-0.940) |  |  | | | | 0.859 (0.800-0.921) | |  |

^a^ Student's t-test
